# Optimization of prostate cancer cell detection using multiplex tyramide signal amplification

**DOI:** 10.1002/jcb.28016

**Published:** 2018-11-02

**Authors:** Sounak Roy, Haley D. Axelrod, Kenneth C. Valkenburg, Sarah Amend, Kenneth J. Pienta

**Affiliations:** ^1^ The Johns Hopkins University, The Krieger School of Arts & Sciences Baltimore Maryland; ^2^ The James Buchanan Brady Urological Institute, Department of Urology, Johns Hopkins University School of Medicine Baltimore Maryland; ^3^ The Cellular and Molecular Medicine Program Johns Hopkins School of Medicine Baltimore Maryland

**Keywords:** detection, immunofluorescence, metastasis, prostate cancer, tumor microarray, tyramide

## Abstract

Approximately 29 000 men die of prostate cancer (PCa) each year in the United States, and 90% to 100% of them are due to incurable bone metastasis. It is difficult to determine (1) when PCa disseminates in the natural history of the disease; (2) where cancer cell disseminates before becoming overt metastatic lesions; and (3) which tumors are aggressive and which are indolent. Tumor tissue and liquid (blood and bone marrow) biopsies provide important information to answer these questions, but significant limitations exist for immunostaining strategies that assess protein expression in these tissues. Classic immunohistochemistry (IHC) assays can typically assess expression of one or two proteins per tissue section. We have developed a novel immunofluorescence staining protocol to detect a panel of seven proteins on PCa tissue from primary tumor biopsies and metastatic lesion autopsy tissue, as well as cancer cells from liquid biopsies. We used a tyramide‐based system to amplify the true signal and optimized the protocol to reduce background signal, thereby boosting the signal‐to‐noise ratio. Any protein‐specific antibody in this protocol can be exchanged for a different validated antibody. This protocol therefore, represents a highly informative and flexible assay that can be used to provide important information about cancer tissue for the purpose of improving detection, diagnosis, and treatment.

## 1 INTRODUCTION

Most of the approximately 29 000 prostate cancer (PCa) deaths predicted in the United States in 2018 will be due to bone metastasis.^1^, ^2^ Notably, most men diagnosed with PCa do not die of their disease; approximately two‐thirds are cured with surgery (radical prostatectomy) or radiation therapy of their primary tumor, diagnosed before metastasis.^3^ However, many men who undergo curative treatment will eventually develop the recurrent disease.^4^, ^5^ While several biological assays exist to appropriately stage PCa,^6^, ^7^, ^8^ it remains difficult to differentiate indolent disease from lethal disease early in its natural history before metastasis and assign treatment strategies based on this information. One possible way to identify different subsets of PCa (ie, indolent vs lethal metastatic) is through immunostaining assays which assess protein expression at a cellular level. Conventional immunohistochemistry (IHC) staining protocols have limited capacity and can only assess one or two proteins at once. Multiplex immunofluorescence (IF) strategies can assess multiple protein markers simultaneously, adding valuable information for the improved clinical staging of cancer.

Several challenges exist in IF‐based detection and analysis of protein expression.^9^ Fluorescent detection can be more sensitive than the chromogenic staining commonly used for clinical evaluation, but higher background signal in IF staining may reduce specificity. Second is the antibody‐independent autofluorescence inherent to formalin‐fixed paraffin‐embedded (FFPE) tissue. Therefore, boosting the signal‐to‐noise ratio to visualize true signal and avoid false signal is critical. For samples with high levels of immune cells (eg, bone marrow), additional false positivity is apparent due to the binding of immune cells to fragment crystallizable (Fc) regions of antibodies. Care must be taken to eliminate this type of false background.^10^ Finally, for IF stains specifically, the number of visible wavelengths that can be visualized simultaneously, are limited due to spectral overlap. Certain microscopes are able to use individual lasers and filters to compensate and subtract overlapping signal, but the typical fluorescent microscope can only visualize four distinct channels at a time. To expand the number of markers that can be assessed together in one sample, there is a need for the ability to perform multiple rounds of staining.^11^

There is a multitude of PCa markers that when combined, would provide more valuable clinical information compared with each expression profile alone.

For example, the epithelial marker pan‐cytokeratin (PanCK) is commonly used to mark cancer cells of epithelial origin, but its expression is lost during epithelial‐to‐mesenchymal transition.^12^, ^13^ Cells that have made this transition are likely more lethal, so it is critical that additional prostate‐specific markers are used to make a complete evaluation. Because these prostate‐specific markers do not always have the same expression pattern throughout disease progression and have high intratumoral and interpatient variability, multiple markers must be assessed simultaneously.^14^

To assess expression of multiple proteins in prostate tumors, we developed a multiplex, multiround IF protocol to visualize expression of eight markers simultaneously: nuclear marker 4′,6‐diamidino‐2‐phenylindole (DAPI), nucleolar marker nucleolin, epithelial marker PanCK, and prostate‐specific markers prostate‐specific antigen (PSA), prostate‐specific membrane antigen (PSMA), androgen receptor (AR), prostein, and alpha‐methylacyl‐CoA racemase (AMACR). This technique may be useful for histopathological analysis of PCa tissue with the goal of assigning patients to a more personalized staging category with a more targeted treatment plan. Moreover, it may be modified to be used with a myriad of antibodies specific to other research questions, enabling for basic science inquiry in any disease state, tissue type, or protein of interest.

## 2 MATERIALS AND METHODS

### 2.1 Preparation of LNCaP cell block for staining control

LNCaP PCa cells (ATCC, Manassas, VA) cultured in Roswell Park Memorial Institute medium (RPMI) containing 10% fetal bovine serum (FBS) and 1% penicillin/streptomycin were centrifuged in a 0.5‐mL tube containing a solid layer of agar and fixed in 4% paraformaldehyde for 24 hours. The cell block was paraffin‐embedded and serial‐sectioned onto plus slides by Johns Hopkins Pathology.

### 2.2 Tissue microarray

PCa tumor microarray slides with 30 tissue cores (0.6‐mm diameter) were obtained from Dr Angelo DeMarzo from the Johns Hopkins Cancer Research Pathology Department. Tumor microarray slides include prostatic tissue cores from four healthy patients (three cores from two patients; two cores from two patients; total = 10) and five patients with primary PCa (three cores from two patients; two cores from two patients; total = 10) and ten healthy nonprostatic tissue cores.

### 2.3 Patient samples

FFPE sections of the 10 patients’ lymph nodes and ten bone metastases were obtained from Johns Hopkins Pathology as part of the Legacy Gift Rapid Autopsy program.

### 2.4 CTC mouse model

LNCaP cells were cultured as above, harvested using nonenzymatic cell dissociation buffer (#13151014; Thermo Fisher Scientific, Waltham, MA), and spiked into bone marrow obtained from killed immunodeficient NSG mice (The Jackson Laboratory, Bar Harbor, ME. The sample was spread on Marienfeld slides before staining. All experiments were approved by the Johns Hopkins Institutional Animal Care and Use Committee.

### 2.5 Rehydration and heat‐induced antigen retrieval

FFPE slides were deparaffinized with Citrisolv and rehydrated with 100%, 95%, and 70% ethyl alcohol (EtOH) for 10 minutes each and then washed in water 2× for 5 minutes. Slides were placed in Antigen Unmasking Solution (Citrate) diluted in distilled water, microwaved at 100% power for 50 seconds followed by 20% power for 20 minutes in microwave‐safe containers and cooled at room temperature for 15 minutes.

### 2.6 First round of optimized staining protocol

All staining steps were performed at room temperature in the dark in a humidity chamber except for subsequent antigen retrieval. Slides were washed in phosphate‐buffered saline (PBS) or PBS with Tween‐20 (PBST) between each step. All primary antibodies were diluted in 5% bovine serum albumin (BSA). Slides were blocked with TrueBlack diluted in 70% EtOH (5 minutes), followed by Image‐iT FX Signal Enhancer (30 minutes), followed by 5% BSA in PBS (30 minutes). Slides were incubated in primary antibody solution (anti‐PSMA, anti‐prostein) for 1 hour. Slides were then incubated in fluorophore‐conjugated secondary antibody solution (goat anti‐rabbit‐AF488 and goat anti‐mouse‐AF555) diluted 1:2500 (45 minutes). Slides were incubated in fluorophore‐conjugated primary antibody solution (anti‐Cytokeratin‐AF647) (1 hour). DAPI (1 mg/ml) was added for 5 minutes. Slides were coverslipped with Prolong Gold Antifade Mountant and cured overnight before manual imaging.

### 2.7 Second round of optimized staining protocol

Following imaging, slides were placed in PBS overnight on a rocker to dislodge coverslips. Heat‐induced antigen retrieval steps were followed as above, then slides were placed in 3% hydrogen peroxide (H_2_O_2_) for 10 minutes. Blocking steps were followed as in the first round of staining. Slides were incubated with the anti‐PSA primary antibody (1 hour) followed by PowerVision horseradish peroxidase (HRP)‐conjugated anti‐rabbit secondary antibody (45 minutes) and then the tyramide‐AF647 working solution (10 minutes in the dark) according to the manufacturer’s instructions. Reaction stop reagent was added directly on the slides. Another round of heat‐induced antigen retrieval was performed. Upon cooling, the anti‐AR primary antibody was added for 1 hour. Secondary antibody and tyramide steps were repeated using tyramide‐AF555 working solution. Finally, slides were incubated in fluorophore‐conjugated antibody solution (antinucleolin‐AF488) (1 hour). Slides were then incubated in DAPI and coverslipped identical to round 1. After overnight curing, slides were manually imaged using the absolute XY coordinates from round 1.

### 2.8 Third round of optimized staining protocol

Coverslip removal, antigen retrieval, and blocking were identical to previous rounds. Anti‐AMACR primary antibody (diluted 1:100) followed by HRP‐conjugated anti‐rabbit secondary and then tyramide AF488. Slides were stained with DAPI, coverslipped, and imaged as described in the first two rounds.

### 2.9 Manual imaging of slides

Slides were imaged using a CoolCube 2‐m monochrome camera (#h_0310‐013‐MS; MetaSystems, Newton, MA), Isis Fluorescence Imaging Platform (V5.8.5; MetaSystems, Newton, MA) using both Zeiss EC Plan‐Neofluar 40×/0.75 M27 and Zeiss Plan‐Achromat objectives. Exposure time (never exceeded 120 ms) and upper and lower thresholds were determined using the sample with the highest fluorescence and were consistent between slides of the same experiment each round. Identical sections of the slide were imaged using absolute *XY* coordinates of the motorized eight‐slide stage which was controlled with a manual movement control system (MMC) (V2.4.5; MetaSystems). DAPI was visualized using 359 nm excitation and 461 nm emission. AF488 was visualized using 495 nm/25 nm excitation and 537 nm/29 nm (wavelength/bandwidth) emission (#49303; Chroma). AF555 was visualized using 550 nm/25 nm excitation and 605 nm/70 nm emission (Filter Set 43 HE; Zeiss). AF647 was visualized using 640 nm/30 nm excitation and 690 nm/50 nm emission (#49009; Chroma).

## 3 RESULTS

### 3.1 Blocking optimization and signal boosting

To block background fluorescence in our cell‐block controls and patient samples, we used an optimized protocol we previously developed which utilizes a combination of TrueBlack, Image‐iT FX Signal Enhancer, and 5% BSA.^10^ To significantly boost the signal up to 200‐fold, we used a tyramide signal amplification protocol. For the three markers that were boosted with tyramide, secondary detection was done using a poly‐HRP‐conjugated secondary antibody so that tyramide initially bound to the HRP and, in the presence of H_2_O_2_, formed a covalent bond with the tyrosine residue of the original antigen. This covalent bond allows the fluorescent signal to remain associated with the tissue during the heat‐induced antigen retrieval steps used to remove antibodies.

This IF protocol was divided into three rounds of staining and imaging separated by rounds of heat‐induced antigen retrieval. Current commercial methods using tyramide are limited by the total number of channels on the microscope. Another inherent limitation of IF is in the inability to use more than one primary antibody made in the same species due to resulting nonspecific binding of secondary antibodies. The ability for tyramide to covalently bind the antigen and remain bound following antigen retrieval allows our protocol to bypass this limitation and use multiple antibodies made in the same species in the same round of staining.

**Figure 1:**
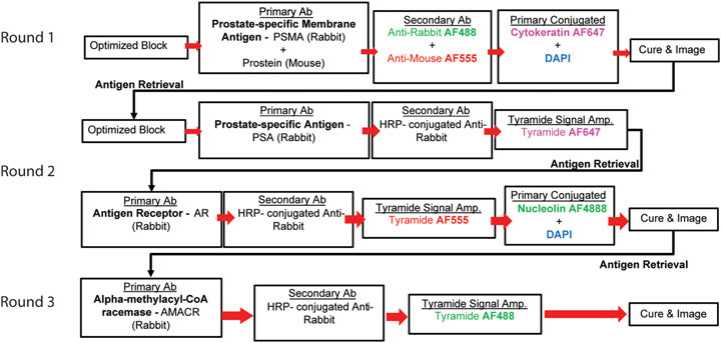
Novel multiplex tyramide signal amplification immunofluorescence protocol. Multiplex tyramide signal amplification can be organized in a flexible protocol divided into three rounds of staining separated by heat‐induced antigen retrieval and imaging. Optimized block includes a combination of TrueBlack, Image‐iT FX Signal Enhancer, and 5% BSA. Anti‐human primary antibodies can be substituted for mouse‐specific or other species‐specific antibodies without requiring alterations to subsequent secondary antibody or tyramide reagents. Curing is an overnight process and imaging is followed by overnight coverslip removal before the subsequent antigen retrieval and blocking steps can begin. Round 2 contains an antigen retrieval step to allow for the usage of two primary antibodies both made in rabbit. Any of the three rounds may combine primary antibodies of the same species given that they are separated by an antigen retrieval step and are paired to different tyramide fluorophores. It is important to note that once a specific tyramide fluorophore has been used that channel may no longer be used in following rounds. BSA, bovine serum albumin; DAPI, 4′,6‐diamidino‐2‐phenylindole; HRP, horseradish peroxidase; PSA, prostate‐specific antigen; PSMA, prostate‐specific membrane antigen

By integrating these techniques, we developed a novel multiplexed protocol that allows clear visualization of up to five prostate‐specific markers plus cytokeratin (CK), nucleolin, and nuclear staining using only a standard four‐channel fluorescent microscope (Figure 1). The goal of this protocol is to offer a robust yet highly flexible method of detecting tissue‐specific cell markers with a higher signal‐to‐noise ratio than current solution, and can be adapted to reliably assess the protein expression in rare cells from liquid biopsies.

### 3.2 FFPE cell‐block staining

**Figure 2:**
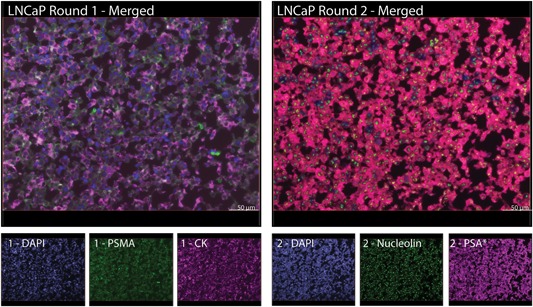
PCa‐specific cell markers are strongly expressed in LNCaP cell blocks. LNCaP cell blocks were stained with two rounds. Separate channels were imaged with maximum exposure times determined by the strongest signal and then merged using the Isis Fluorescence Imaging software, MetaSystems, Newton, MA. Channels boosted with tyramide in all figures are indicated with an asterisk. Scale bar = 50 μm. CK, cytokeratin; DAPI, 4′,6‐diamidino‐2‐phenylindole; PCa, prostate cancer; PSA, prostate‐specific antigen; PSMA, prostate‐specific membrane antigen

LNCaP cell blocks were stained as positive controls to optimize imaging. DAPI, PSMA, and CK were expressed strongly during the first round of imaging, while DAPI, nucleolin, and PSA expression were captured in round 2 (Figure 2). Negative control slides with no primary antibody but otherwise identical staining were imaged in parallel to ensure no nonspecific binding of secondary antibodies occurred.

### 3.3 Tumor microarray staining

**Figure 3:**
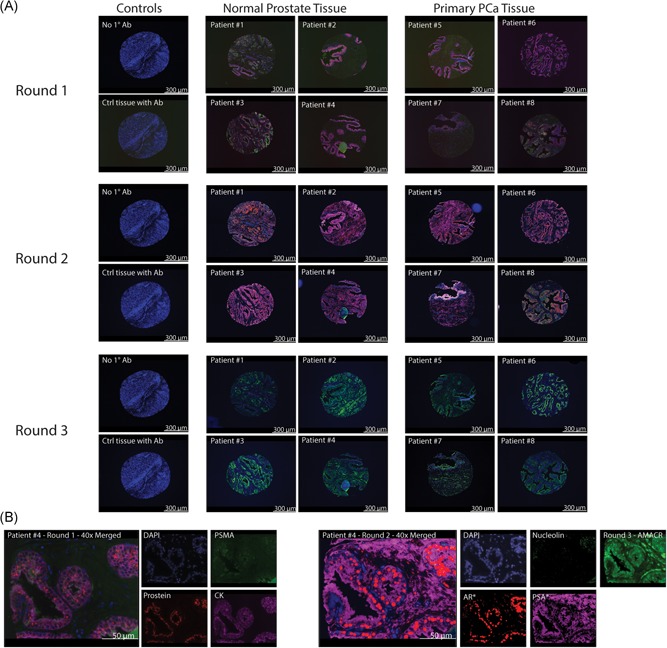
Multiplex tyramide signal amplification allows for differentiation between control and prostate tissue. A, TMA sections were stained with three rounds in the order outlined in Figure 1 and imaged at ×10 using the Zeiss objective and channels were merged using Isis Fluorescence Imaging Software. One representative core from each of the eight patients was chosen, imaged after each round, and compared to representative cores from each of the healthy nonprostatic tissues. The difference in staining intensity is apparent for each round indicating a robust ability for our protocol to differentiate prostate‐specific tissue. Scale bar = 300 μm. B, The tissue core for patient #4 was imaged at ×40 to demonstrate the high degree of specificity of antibody binding in distinctly localized regions of cells in the tissue. Nucleolin stains in prostate tissue emit a single signal per cell. Scale bar = 50μm. AMACR, alpha‐methylacyl‐CoA racemase; CK, cytokeratin; DAPI, 4′,6‐diamidino‐2‐phenylindole; PCa, prostate cancer; PSA, prostate‐specific antigen; PSMA, prostate‐specific membrane antigen; TMA, tumor microarray

A tumor microarray was stained for three rounds with imaging after each round. A separate slide was stained simultaneously while excluding primary antibodies to serve as a negative control. Tumor microarray slides include healthy nonprostatic tissue cores and prostatic tissue cores from healthy patients and PCa patients. DAPI, PSMA, Prostein, and CK staining during round 1 were positive in 9 out of 10 PCa patient prostatic cores and localized to the nucleus, cell‐surface, golgi, and cytoplasm, respectively. DAPI, nucleolin, AR, and PSA staining during round 2 were positive in 10 out of 10 PCa patient prostatic cores and localized to the nucleus, nucleolus, nucleus, and cell‐surface, respectively. Control, nonprostatic tissue only stained for DAPI and otherwise demonstrated no nonspecific binding after any of the three rounds (Figure 3). Positive AMACR staining was observed in 6 out of 10 healthy prostate tissue cores and 10 out of 10 primary PCa cores (Figure 3).

### 3.4 Human lymph node and bone metastases

**Figure 4:**
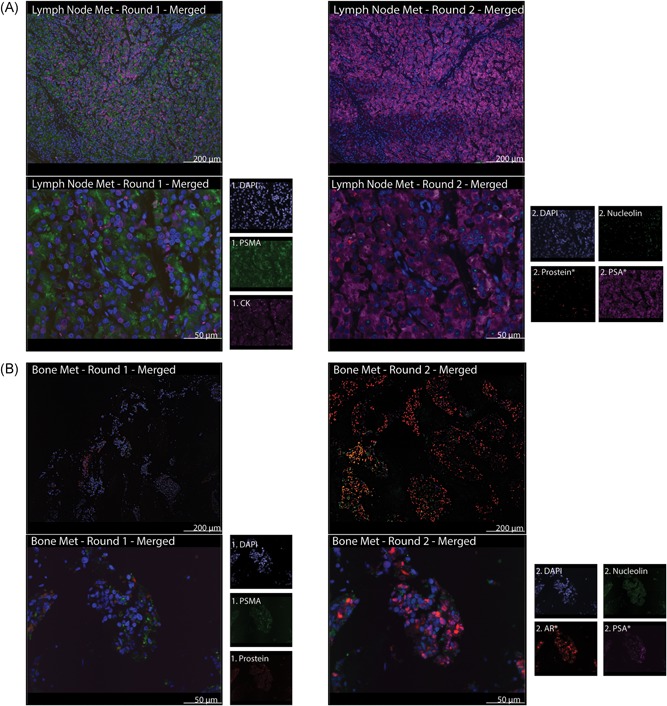
A novel panel of six markers common in PCa can be detected in human lymph node and bone metastases. A, Tissue sections of human patient lymph node Mets were stained with two rounds and imaged at ×10 (above) and ×40 (below). Individual channels are shown for the ×40 images to demonstrate distinct localizations of prostate‐specific markers while ×10 images demonstrate that staining was not limited to edge effects or restricted to small regions of the tissue section. ×10 image scale bars = 200 μm and ×40 image scale bars = 50 μm. B, Tissue sections of human bone metastases were stained with two rounds and also imaged at ×10 (above) and ×40 (below). All antibodies boosted with tyramide are marked with an asterisk. Only in nucleolin stains of cells in bone metastases sections is there two distinct signals per cell as visible from the 488 nm (green) channel. ×10 image scale bars = 200 μm and ×40 image scale bars = 50 μm. AR, androgen receptor; CK, cytokeratin; DAPI, 4′,6‐diamidino‐2‐phenylindole; PCa, prostate cancer; PSA, prostate‐specific antigen; PSMA, prostate‐specific membrane antigen

Ten patient FFPE lymph node metastasis samples and 10 patient FFPE bone metastases were stained with DAPI, PSMA, AR, and CK for round 1 and DAPI, PSA, prostein, and nucleolin for round 2. One slide each of lymph node metastasis and bone metastasis samples served as an unstained negative control. DAPI, PSMA, and CK were expressed strongly in the human lymph node metastases and localized in the nucleus, cell‐surface, and cytoplasm, respectively. AR staining was indistinguishable from PSMA signal due to overlapping localization. DAPI, PSA, prostein, and nucleolin, all were stained strongly positive in lymph node metastases in round 2 with distinct localization in the nucleus, cell‐surface, golgi, and nucleolus, respectively (Figure 4A). DAPI, PSMA, and prostein in FFPE human bone metastases stained strongly positive in round 1 with distinct localizations in the nucleus, cell‐surface, and golgi, respectively. DAPI, nucleolin, AR, and PSA, all were stained strongly positive in round 2 with localizations in the nucleus, nucleolus, and cell‐surface, respectively (Figure 4B). AF555 tyramide amplification of AR staining in round 2 resulted in strong signal and facilitated visualization.

### 3.5 Detection of LNCaP cells in mouse bone marrow

Our novel multiplex tyramide signal amplification technique was further optimized to visualize human LNCaP cells spiked into bone marrow from immunocompromised mice. Using a staining protocol design as described above allow for the visualization of PSA (round 1), AR (round 2), and human leukocyte antigen (HLA) with DAPI nuclear stain (round 3) with heat‐induced antigen retrieval in‐between rounds. Following staining, we detected a cluster of LNCaP cells with localized PSA and HLA staining. No nonspecific binding was detected after three rounds of staining in the unspiked bone marrow.

## 4 DISCUSSION

Early detection of recurrent PCa could prevent many deaths that occur due to incurable disease progression in secondary sites such as bone. Promising strategies for rare cell detection include IF staining of blood and bone marrow. However, sensitive and specific detection of these cells requires a robust staining protocol combined with the ability to use multiple prostates and cancer‐specific markers. Our optimized protocol can detect up to seven unique markers plus a nuclear stain, allowing for significant flexibility in the choice of markers and the ability to boost the signal of low expressed proteins using tyramide. The manual imaging method we used can also be adapted to include quantification of regions positive for specific markers to compare within tissue samples from a single patient or between different patient samples to develop stronger conclusions about disease prognosis. While only four channels are required to image all seven markers plus the nuclear stain, the incorporation of a fifth channel to detect 800 nm emission spectra would allow for up to three additional unique markers. Furthermore, using the 405 nm channel with tyramide signal amplification in round 3 that was reserved for DAPI nuclear staining in the first two rounds allows an additional marker to be incorporated. Altogether, our novel protocol can be expanded to a theoretical maximum of 11 unique markers plus a nuclear stain.

Standard multiplex IF is necessarily restricted by the number of available antibodies host‐species, limiting both total number of markers and reducing the number of available antibodies for a particular marker (ie, only one antibody can be anti‐rabbit, one anti‐mouse, etc). Our multiplex approach allows for multiple antibodies with the same host‐species to be used during the same round of staining, thus enabling simultaneous visualization. An additional flexibility of the protocol is the ability to seamlessly interchange antibodies. The fluorophore‐conjugated secondary antibody, tyramide signal amplification, and heat‐induced antigen retrieval can be kept consistent, where only the primary antibodies have to be replaced with antibodies specific for other tissue types or even species.

Using our multiround protocol, we observed the consistent staining of each marker in LNCaP cell blocks. The variability in staining intensity among patient lymph node and bone metastases can therefore, be attributed with variability in protein expression. Future staining of larger patient cohorts would allow for the development of a robust database that predicts disease outcomes using expression profiles of prostate‐specific markers. Imaging of the lymph node and bone metastases revealed a dramatic change in protein expression profile. For all PCa metastasis stained, bone metastases had increased nucleolar number seen as two distinct signals following primary‐conjugated antinucleolin staining compared with a single fluorescent area in the nucleus that is observed in lymph node metastases. Increase in size and number, nucleoli has often been shown to be a hallmark of PCa progression.^15^, ^16^ The consistency of the change in expression supports the eventual use of our protocol to not only detect PCa cells early in disease progression but to also quantify expression throughout various stages and secondary sites (Table 1).

**Table 1:** Reagents used for IF multiplex tyramide signal amplification

Reagent	Company	Catalog
10× PBS pH 7.4	Quality Biological, Gaithersburg, MD	119‐069‐131CS
Citrisolv clearing agent	Fisher Scientific, Pittsburgh, PA	22143975
Tween‐20	Sigma, St. Louis, MO	P9416
Antigen Unmasking Solution (Citrate)	Vector Laboratories, Burlingame, CA	H‐3300
BSA	Fisher Scientific, Pittsburgh, PA	BP1600‐1
TrueBlack Lipofuscin Autofluorescence Quencher	ChemoMetec, Bohemia, NY	23007
Image‐iT FX Signal Enhancer	Thermo Fisher, Waltham, MA	I36933
ProLong Gold Antifade Mountant	Thermo Fisher	P36930
Alexa Fluor 647 Tyramide SuperBoost Kit, goat anti‐rabbit IgG	Thermo Fisher	B40926
Alexa Fluor 555 Tyramide SuperBoost Kit, goat anti‐rabbit IgG	Thermo Fisher	B40923
Alexa Fluor 488 Tyramide SuperBoost Kit, goat anti‐rabbit IgG	Thermo Fisher	B40922
Anti‐PSMA	Cell Signaling, Danvers, MA	12702
Anti‐prostein (P501S)	Agilent, Santa Clara, CA	M3615
Anti‐cytokeratin AF647 (CK)	Biolegend, San Diego, CA	628604
Anti‐PSA	Cell Signaling	5365
Anti‐AR	Cell Signaling	5153
Antinucleolin‐AF488	Abcam, Cambridge, UK	Ab154028
Anti‐AMACR monoclonal Ab (13H4)	Thermo Fisher	MA5‐14576
Goat anti‐rabbit IgG (H + L) AF488	Thermo Fisher	A11034
Goat anti‐mouse IgG (H + L) AF555	Thermo Fisher	A32727
PowerVision Poly‐HRP anti‐rabbit secondary antibody	Leica Biosystems, Buffalo Grove, IL	PV6119
Moisture chamber	Evergreen Scientific, Rancho Dominguez, CA	240‐9020‐Z10
Microwave‐safe container	PerkinElmer, Waltham, MA	STJAR4
Vertical glass staining dish	Fisher Scientific	08‐815
1250 W microwave	Panasonic, Kadoma, Osaka, Japan	NN‐T945SFX

AMACR, alpha‐methylacyl‐CoA racemase; AR, androgen receptor; CK, HRP, PSA, prostate‐specific antigen; PSMA, prostate‐specific membrane antigen.

The purpose of this technique was to significantly improve the signal‐to‐noise ratio of PCa detection using IF and to this end we have developed a robust and easily modifiable multiplex protocol that uses tyramide signal amplification to detect a panel of seven prostate and PCa‐specific markers in cell blocks, TMA slides, and FFPE lymph node and bone metastases. We have also begun preliminary optimization of multiround detection of rare cells in liquid cell biopsies. Observations also indicate that this protocol can be used to better understand the significance of expression patterns in later‐stage disease.

## Supporting information

Supplementary information
