# Supplementary material for: Optimization of prostate cancer cell detection using multiplex tyramide signal amplification
Source: J Cell Biochem. 2018 Nov 2;120(4):4804–12. doi: 10.1002/jcb.28016 (PMC6519224; doi:10.1002/jcb.28016)
Supplement: Supplementary file 1 — Supplementary information [file JCB-120-4804-s001.docx]

**Multiplex multi-round tyramide signal amplification – for FFPE Slides**

Slides

TMA 1107 2x slides

Materials

1. Citrate buffer (1:100 Antigen Unmasking Solution – stored in 4C deli fridge)
2. TrueBlack (stored in RT next to Haley – in clear tubes Biotium CAT#23007)
3. Image-iT FX Signal Enhancer (stored in oversized reagents box – 4C deli fridge)
   1. Clear Dropper – Invitrogen Lot#1922327
4. 5% BSA
   1. In a 50mL Conical in 4C deli fridge – near oversized reagents box
   2. To remake: 5g BSA powder (container with blue lid in bottom shelf of 4C deli fridge) in 100 mL PBS
5. DAPI (not pre-suspended in mounting media)
   1. 1000x stock stored in Valkenburg box (small white -20C freezer)
6. DAPI-free mounting media
   1. In Brown tube in Haley’s bench on shelf at RT
   2. Anti-fade with label “No DAPI”
7. 100X Tyramide Stock Solution
   1. Dissolve Alexa Fluor Tyramide reagent (Component C1) in 150 μL (for 150 slides) of DMSO (component E). Invert the vial several times to dissolve any Tyramide that might coat the sides of the vial.
   2. Store stock at 2-8°C up to 6 months.
8. 100X H_2_O_2_ solution
   1. Add 1 drop (50 μL) of Hydrogen Peroxide Solution (Component C2) to 1 mL of distilled water.
9. 1X Reaction Buffer
   1. Add 1 drop (approximately 50μL) of 20X reaction buffer to 1 mL of distilled water.
   2. Prepare only on day of use. Tris Buffer (pH 7.4) can be substituted for reaction buffer)
10. Tyramide Working Solution


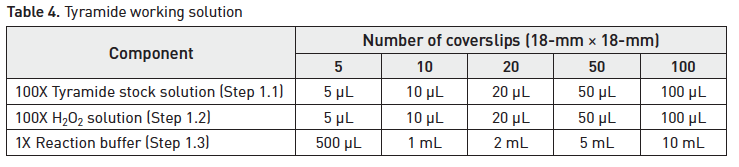


1. Reaction Stop Reagent Stock
   1. Add 1.45 mL of 95% ethanol to one vial of Reaction Stop Reagent (Component D).
   2. Vortex vial to dissolve stop reagent on sides of bottle.
   3. Can store in -20°C for 6 months.
2. Stop Reagent working solution
   1. Dilute Stop Reagent Stock **1:11** in PBS.
   2. Prepare only on day of use.

**Antibodies – Round 1**

1. Cell surface: PSMA(rabbit) + CK(mouse)
2. Cytoplasm (Golgi): Prostein (mouse)
3. DAPI (stain)

Whole & F(ab) secondary antibodies are stored in 2° IF Ab box in 4C deli fridge

**Antibodies – Round 2**

1. Cell surface: PSA (rabbit)
2. Nuclear: AR
3. Nuclear: Nucleolin

**Antibodies – Round 3**

1. Cell surface: AMACR (rabbit)

Methods

**Day 1**

A. Rehydrate slides

1. Citrisolv for 15 min
2. 100% EtOH for 10 min
3. 95% EtOH for 10 min
4. 70% EtOH for 10 min
5. Wash in water 2x 5 min

B. Antigen Retrieval – Round 1

1. **Microwave** for 20 minutes – in Citrate buffer
   1. 100% power for 50 seconds then 20% power for 15 min
2. Cool for 15-20 minutes on bench
3. Wash in water 2x 5min
4. Wash in PBST 1x 5min
5. Wash in PBS 2x 3min

C. Staining – Round 1

1. Block with TrueBlack by adding fresh (1:20 diluted) TrueBlack diluted in 70% EtOH for 1min at RT
2. Wash in PBS 2x 3min
3. Wash in PBST 1x 3min
4. Block with Image-iT for 30min at RT (4-5 drops on a slide)
5. Block with 5% BSA for 30min at RT
6. Add primary antibody diluted in 5% BSA for 1hr at RT
7. Wash in PBST 3x 3min
8. Add whole secondary antibodies diluted in PBS at 1:2500 for 45min at RT
   1. WHOLE = for any immune marker (CD45, CD11-b) (1:2500)
   2. Secondary antibodies expire after 30 days
9. Wash in PBST 3x 3min
10. Add primary **conjugated** antibody diluted in 5% BSA for 1hr at RT
    1. This is the Pan-CK Ab for Round 1
11. Wash in PBST 3x 3min
12. Add DAPI diluted 1:1000 in PBS for 5min at RT
13. Wash in PBST 3x 3min
14. Mount coverslip using DAPI-free mounting media
15. Let slides cure overnight

**Day 2**

*Imaging Round 1* - Metafer: Isis Fluorescence Imaging Platform

D. Take off Coverslip

1. Put in PBS
2. Shake overnight (might be a shorter time for smaller coverslips)

**Day 3**

E. Antigen Retrieval – Round 2

1. **Microwave** for 20 minutes – in Citrate buffer
   1. 100% power for 50 seconds then 20% power for 15 min
2. Cool for 15-20 minutes on bench
3. Wash in water 2x 5 min
4. Incubate in 3% Hydrogen Peroxide (1:10 in water) 10 min RT
   1. Hydrogen peroxide only because of HRP containing antibody
5. Wash in water 2x 5 min
6. Wash in PBS 2x 5 min
   1. (do not use PBST before TrueBlack b/c Tween will mess up TrueBlack)

F. Staining – Round 2

1. Block with TrueBlack by adding fresh 20x TrueBlack diluted in 70% EtOH for 1min at RT
2. Wash in PBS 2x 3min
3. Wash in PBST 1x 3min
4. Block with Image-iT for 30min at RT
5. Block with 5% BSA for 30min at RT

G. Tyramide – HRP Rabbit

1. Add primary antibody diluted in 5% BSA for 1hr at RT
2. Wash in PBST 3x 3min
3. Add HRP-conjugated secondary antibody 45min at RT – Power vision HRP conjugated Rabbit (4-5 drops – white bottle in 4C)
4. Wash in PBST 2x 5min
5. Wash in PBS 1x 5min
6. Incubate in fluorophore-conjugated **Tyramide** working solution for 10 min at RT in **dark**
7. Add STOP solution (same vol. as Tyramide working solution) directly to Tyramide for few secs, then decant and place into PBS
8. Wash in PBS 3x 3min

H. Antigen Retrieval

1. **Microwave** for 20 minutes – in Citrate buffer
   1. 100% power for 50 seconds then 20% power for 15 min
2. Cool for 15-20 minutes on bench

I. Tyramide – HRP Rabbit

1. Add primary antibody diluted in 5% BSA for 1hr at RT
2. Wash in PBST 3x 3min
3. Add HRP-conjugated secondary antibody 45min at RT – Power vision HRP conjugated Mouse (4-5 drops – white bottle in 4C)
4. Wash in PBST 2x 5min
5. Wash in PBS 1x 5min
6. Incubate in fluorophore-conjugated **Tyramide** working solution for 10 min at RT in **dark**
7. Add STOP solution (same vol. as Tyramide working solution) directly to Tyramide for few secs, then decant and place into PBS
8. Wash in PBS 3x 3min
9. Wash in water 2x 5 min
10. Add primary **conjugated** antibody diluted in 5% BSA for 1hr at RT
    1. This is the Nucleolin Ab for Round 2
11. Wash in PBST 3x 3min
12. Add DAPI diluted 1:1000 in PBS for 5min at RT
13. Wash in PBST 3x 3min
14. Mount coverslip using DAPI-free mounting media
15. Let slides cure overnight

**Day 4**

*Imaging Round 2* - Metafer: Isis Fluorescence Imaging Platform

J. Take off Coverslip

1. Put in PBS
2. Shake overnight (might be a shorter time for smaller coverslips)

**Day 5**

K. Antigen Retrieval – Round 2

1. **Microwave** for 20 minutes – in Citrate buffer
   1. 100% power for 50 seconds then 20% power for 15 min
2. Cool for 15-20 minutes on bench
3. Wash in water 2x 5 min
4. Incubate in 3% Hydrogen Peroxide (1:10 in water) 10 min RT
   1. Hydrogen peroxide only because of HRP containing antibody
5. Wash in water 2x 5 min
6. Wash in PBS 2x 5 min
   1. (do not use PBST before TrueBlack b/c Tween will mess up TrueBlack)

L. Staining – Round 2

1. Block with TrueBlack by adding fresh 20x TrueBlack diluted in 70% EtOH for 1min at RT
2. Wash in PBS 2x 3min
3. Wash in PBST 1x 3min
4. Block with Image-iT for 30min at RT
5. Block with 5% BSA for 30min at RT

M. Tyramide – HRP Rabbit

1. Add primary antibody diluted in 5% BSA for 1hr at RT
2. Wash in PBST 3x 3min
3. Add HRP-conjugated secondary antibody 45min at RT – Power vision HRP conjugated Rabbit (4-5 drops – white bottle in 4C)
4. Wash in PBST 2x 5min
5. Wash in PBS 1x 5min
6. Incubate in fluorophore-conjugated **Tyramide** working solution for 10 min at RT in **dark**
7. Add STOP solution (same vol. as Tyramide working solution) directly to Tyramide for few secs, then decant and place into PBS
8. Wash in PBS 3x 3min
9. Add DAPI diluted 1:1000 in PBS for 5min at RT
10. Wash in PBST 3x 3min
11. Mount coverslip using DAPI-free mounting media
12. Let slides cure overnight

**Day 6**

*Imaging Round 3* - Metafer: Isis Fluorescence Imaging Platform

**Decoding Colors**

405 – blue (DAPI)

488 – green

555 – red

647 – cy5 (pink)

**Round 1 – use “whole” secondaries in Goat**

*use Mouse IgG instead of IgG1/IgG2a/2b

| **Antibody** | **Company** | **Catalog#** | **Species** | **Conjugation** | **Secondary detection** | **Dilution** |
| --- | --- | --- | --- | --- | --- | --- |
| PSMA (surface) | Cell Signaling | 12702 | Rabbit | None | Goat anti-rabbit AF488 | Primary: 1:100 |
| Prostein – P501S (cytoplasm – Golgi spots) | Dako/Agilent | M3615 | Mouse | None | Goat anti-mouse IgG AF555 | Primary: 1:100 |
| Pan-CK C11 clone (cytoplasm) | Biolegend | 628604 | Mouse | AF647 |  | Primary: 1:100 |

**Round 2 – Tyramide**

| **Antibody** | **Company** | **Catalog#** | **Species** | **Conjugation** | **Secondary detection** | **Dilution** |
| --- | --- | --- | --- | --- | --- | --- |
| PSA (surface) | Cell Signaling | 5365 | Rabbit | None | AF647  SuperBoost  Tyramide | Primary: 1:100 |
| AR (nuclear) | SantaCruz | Sc-7305 | Mouse | None | AF555  SuperBoost Tyramide | Primary: 1:100 |
| Nucleolin – (nuclear) | Abcam | Ab154028 | Mouse | AF488 |  | Primary: 1:100 |

**Round 3 – Tyramide**

| **Antibody** | **Company** | **Catalog#** | **Species** | **Conjugation** | **Secondary detection** | **Dilution** |
| --- | --- | --- | --- | --- | --- | --- |
| AMACR (surface) |  |  |  | None | AF488  SuperBoost  Tyramide | Primary: 1:100 |
